# Neural Network Connectivity Following Opioid Dependence is Altered by a Common Genetic Variant in the µ-Opioid Receptor, *OPRM1* A118G

**DOI:** 10.1523/JNEUROSCI.1492-23.2023

**Published:** 2024-02-07

**Authors:** Yihan Xie, Julia K. Brynildsen, Kyle Windisch, Julie A. Blendy

**Affiliations:** ^1^Department of Systems Pharmacology and Translational Therapeutics and Perelman School of Medicine, University of Pennsylvania, Philadelphia 19104, Pennsylvania; ^2^Department of Bioengineering, School of Engineering and Applied Science, University of Pennsylvania, Philadelphia 19104, Pennsylvania

**Keywords:** FOS, mice, morphine, neural networks, *Oprm1*, SNP

## Abstract

Opioid use disorder is a chronic, relapsing disease associated with persistent changes in brain plasticity. A common single nucleotide polymorphism (SNP) in the µ-opioid receptor gene, *OPRM1* A118G, is associated with altered vulnerability to opioid addiction. Reconfiguration of neuronal connectivity may explain dependence risk in individuals with this SNP. Mice with the equivalent *Oprm1* variant, A112G, demonstrate sex-specific alterations in the rewarding properties of morphine and heroin. To determine whether this SNP influences network-level changes in neuronal activity, we compared FOS expression in male and female mice that were opioid-naive or opioid-dependent. Network analyses identified significant differences between the AA and GG *Oprm1* genotypes. Based on several graph theory metrics, including small-world analysis and degree centrality, we show that GG females in the opioid-dependent state exhibit distinct patterns of connectivity compared to other groups of the same genotype. Using a network control theory approach, we identified key cortical brain regions that drive the transition between opioid-naive and opioid-dependent brain states; however, these regions are less influential in GG females leading to sixfold higher average minimum energy needed to transition from the acute to the dependent state. In addition, we found that the opioid-dependent brain state is significantly less stable in GG females compared to other groups. Collectively, our findings demonstrate sex- and genotype-specific modifications in local, mesoscale, and global properties of functional brain networks following opioid exposure and provide a framework for identifying genotype differences in specific brain regions that play a role in opioid dependence.

## Significance Statement

Opioid use disorder is moderately heritable, and the common µ-opioid receptor variant (*OPRM1* A118G) has been repeatedly associated with this disease. Opioid use liability is often higher in individuals with a history of chronic exposure and can be moderated by this single nucleotide polymorphism (SNP). Using a mouse model of the *Oprm1* SNP, our work revealed opioid-induced differences in network connectivity between sexes and opioid dependence states in AA and GG *Oprm1* mice. We also identified six (predominantly cortical) brain regions that strongly influence the transition to an opioid-dependent brain state. These data suggest potential brain regions that may be targeted using noninvasive therapeutic approaches such as repetitive transcranial magnetic stimulation and could be useful to inform personalized treatment.

## Introduction

Opioid use disorder (OUD) is a chronic relapsing brain disease that accounted for a staggering 70% of over 100,000 drug overdose deaths in the USA from 2021 to 2022 (Commonwealth Fund, 2022; Cook, 2022). Genetic variants, such as single nucleotide polymorphisms (SNPs), have been associated with OUD susceptibility (Blackwood and Cadet, 2021). A commonly reported SNP in the gene encoding the µ-opioid receptor (*OPRM1* A118G SNP; rs1799971) has an amino acid replacement of asparagine (N) to aspartic acid (D) at an N-glycosylation site of the receptor resulting in reduced receptor expression (Huang et al., 2012; Browne et al., 2017). This SNP is highly prevalent in both Caucasian (8–30%) and Asian (50%) populations (Taqi et al., 2019). The presence of the G allele is associated with reduced receptor expression (Zhang et al., 2005, 2020; Mague et al., 2009) and blunted sensitivity to opioids (Mahmoud et al., 2011; Robinson et al., 2015; Popova et al., 2019). However, the influence of this SNP on brain network dynamics mediating OUD risk has yet to be elucidated.

Compulsive opioid use is a disorder of distributed brain circuits that can be modeled as networks (Bassett et al., 2018). Tools drawn from the branch of mathematics known as graph theory show promise in characterizing circuit disorders across humans and animal models (Braun et al., 2018). For instance, graph theory metrics can be used to assess network efficiency, how easily information can be transferred between brain regions (Achard and Bullmore, 2007), and centrality, or the relative importance of each region in the network (Joyce et al., 2010).

Whereas graph theory metrics enable descriptive characterizations of brain networks, network control theory can provide insight into causal processes underlying changes in brain state across time. Within a network control theory framework, neural activity patterns under different conditions can be understood to represent brain “states.” At a given time point, the brain state depends upon a combination of its previous activity state, its interregional structural connections, and external inputs. The range of accessible brain states can be defined in terms of an energy landscape, in which low-energy brain states are represented as minima, and higher-energy, more difficult-to-reach states are represented as peaks (Gu et al., 2018). In the present study, control energy could be understood to reflect the ease of transitioning to an opioid-dependent brain state.

To better understand the complex relationship between genetics and neural connectivity, we examined functional brain network topology and neural dynamics in a murine model of the *OPRM1* A118G SNP (*Oprm1* A112G; referred to throughout as AA or GG mice). To investigate how this SNP impacts the brain's functional architecture, we generated network models by quantifying the expression of the immediate early gene FOS following acute or chronic opioid exposure in both male and female AA and GG mice. FOS, a well-established neuronal activity marker, can indicate dynamic adaptive changes in response to a broad range of cellular stimuli (Bullitt, 1990; Chung, 2015; Gallo et al., 2018), including opioids (Gogas et al., 1991; Ferguson et al., 2004; David et al., 2007; Alvarez-Bagnarol et al., 2022; Jerzemowska et al., 2022). We constructed FOS correlation networks and assessed their local, mesoscale, and global properties using graph theory metrics.

To determine how the *Oprm1 A112G* SNP influences brain network dynamics during opioid dependence, we used a network control theory approach. We identified six key brain regions (five cortical and one thalamic) that strongly influence the energetic cost of transitioning to an opioid-dependent brain state. Notably, in GG females, these regions exhibit a substantial reduction in their influence. In addition, by weighting the control inputs to each region according to the distribution of opioid receptors, we determined that the spatial distribution of µ-opioid receptors enables a lower-cost state transition relative to the distribution of either δ- and κ-opioid receptors.

While GG male mice show differences in functional network topology in both opioid-naive and opioid-dependent states compared to AA counterparts, the GG females in the dependent state show significant differences even from the GG males. No sex differences are evident in the AA mice under these conditions. Taken together, our results demonstrate a novel role for the *Oprm1* A112G SNP in modulating how the brain dynamically transitions between opioid-naive and opioid-dependent brain states. These characterizations of network-level adaptations in response to opioid exposure implicate an important sex × genotype interaction that has gone unrecognized in many GWAS studies to date.

## Materials and Methods

### Animals

Adult (8–18 weeks, average age = 13.64) C57BL/6 mice (76 in total, *n* = 6 or 8 in each treatment group) were housed on a 12 h light/dark cycle.

### Drug administration

**Figure 1.**
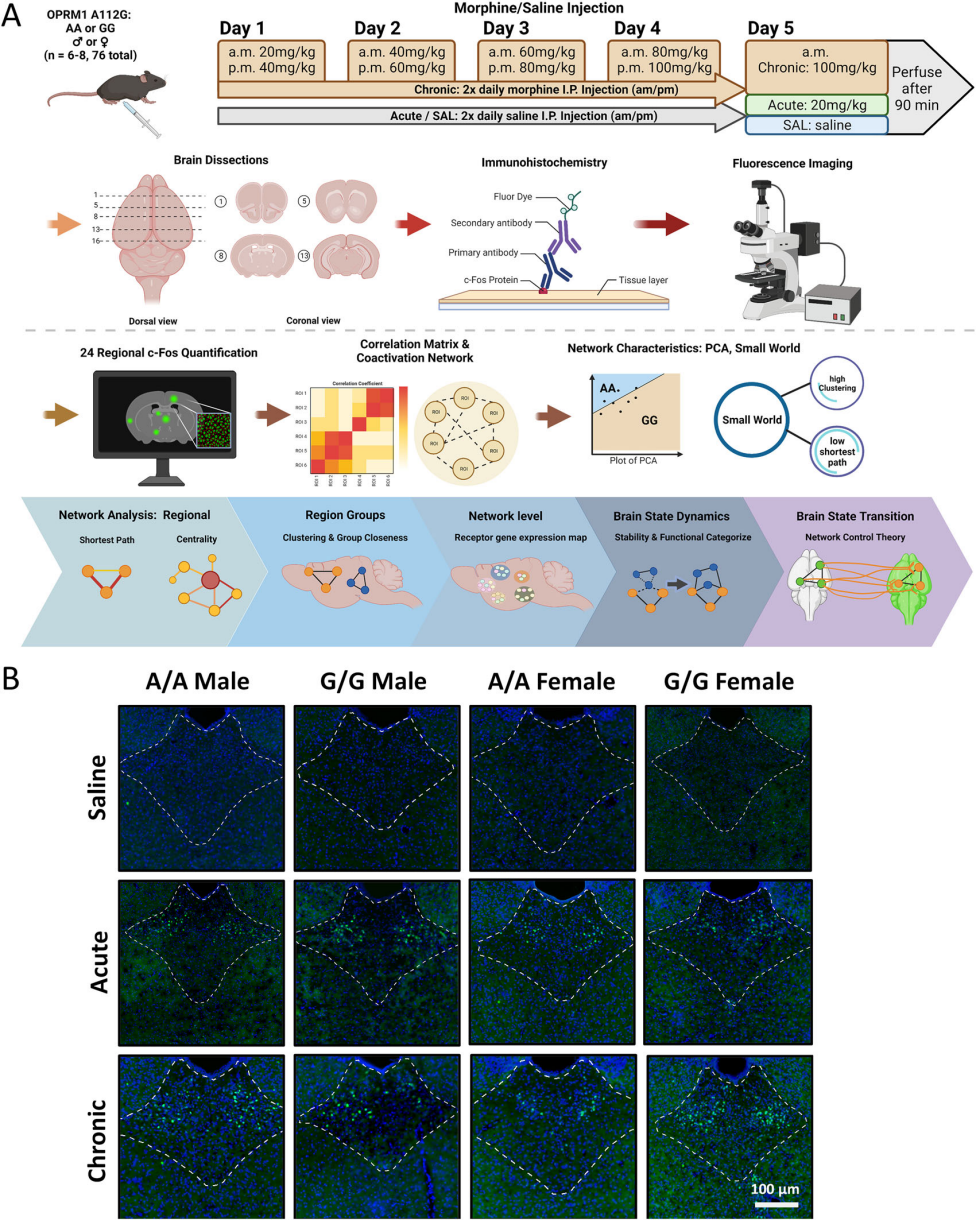
Experimental and analysis paradigm. ***A***, A diagram of the experimental and analysis design. Mice receiving saline, acute, and chronic injections of morphine were perfused, and FOS expression was captured in each of the 24 regions. Created by Biorender.com with an approved license (SQ25JBE6LU). ***B***, An example of FOS expression for each experimental group in paraventricular nucleus of thalamus counterstained with DAPI (PVT).

Male and female mice with *Oprm1* AA or GG alleles were randomly assigned to one of three treatment groups: Saline (0.9%), acute morphine, or chronic morphine. Animals in the saline group received twice-daily intraperitoneal injections of saline over 5 d; the acute morphine group received twice-daily saline on days 1–4 and a single intraperitoneal injection of 20 mg/kg morphine on day 5; animals in the chronic morphine group received escalating doses of intraperitoneal morphine (20–100 mg/kg) twice daily over 5 d (Fig. 1*A*). This escalating dose paradigm is widely used to induce morphine dependence in mice (Maldonado et al., 1996; Brynildsen et al., 2020). Morphine sulfate was obtained from the NIDA Drug Supply Program and dissolved in 0.9% saline. Body weight was measured each day before the morning injection.

### FOS immunohistochemistry

After 90 min since the last injection of morphine or saline, mice were anesthetized with a 50 mg/kg intraperitoneal injection of sodium pentobarbital. Mice were then perfused with 50 ml 1× phosphate-buffered saline (1×PBS, 0°C) followed by 50 ml 4% paraformaldehyde (PFA, 0°C). Brains were post-fixed 48 h in 4% PFA (4°C) and cryoprotected in 30% sucrose (1×PBS-30% sucrose, 4°C). Brains were sectioned on a cryostat, and 30 µM sections were preserved in 1×PBS (1×PBS-0.1% sodium azide) at 4°C. Twelve representative sections were selected based on the Coronal Mouse Brain Atlas from the Allen Institute and were mounted on slides. Mounted sections then underwent 4 × 10 min 1× PBS wash and were blocked in 5% normal goat serum-1×PBS-0.3%Triton X-100 (NGS-PBS-TX) for 60 min. Sections were incubated in rabbit anti-c-Fos primary antibody (1:300 dilution in NGS-PBS-TX, RT; cell signaling) overnight. Samples underwent a second round of 4 × 10 min 1× PBS washes and were incubated for 1 h at RT in goat anti-rabbit Alexa Fluor 488 secondary antibody (1:1,000 dilution). The slides were washed (4 × 10 min in 1× PBS) and covered with DAPI Fluor mount prior to imaging.

### Fluorescence imaging

All slides were imaged on a Keyence BZ-X810 fluorescence microscope with 12 µm capturing depth. The following 24 regions were selected according to our prior paper (Brynildsen et al., 2020) characterizing opioid dependence states: dorsal anterior cingulate area (dACA/ACAd), ventral anterior cingulate area (vACA/ACAv), dorsal agranular insular (AId), ventral agranular insular (AIv), claustrum (CLA), caudoputamen (CP), nucleus accumbens (NAc), bed nucleus of stria terminalis (BNST), ventral pallidum (PALv), central amygdala (CeA), basolateral amygdala (BLA), dentate gyrus (DG), globus pallidus external (GPe), globus pallidus internal (GPi), infralimbic area (ILA), medial hebenula (MH), lateral hebenula (LH), lateral hypothalamus (LHA), paraventricular nucleus of thalamus (PVT), periaqueductal gray (PAG), prelimbic area (PL), ventral tegmental area (VTA), substantial nigra compact part (SNc), substantial nigra reticular part (SNr).

### Quantification of FOS expression

All images were adjusted for brightness and converted to a gray scale (8 bit). ROIs were defined by comparison to reference sections in the Allen Mouse Brain Reference Atlas. Images were auto-thresholded to enable optimal identification of FOS-expressing cells, and the Analyze Particle tool was used to quantify FOS-expressing cells. Damaged sections were manually marked and excluded during data processing. We also estimated the true distribution of expression using bootstrapping, where random frequencies (*N* specified in each result section) of original data were taken and averaged (Singh and Xie, 2010).

### Quantification validity

**Figure 2.**
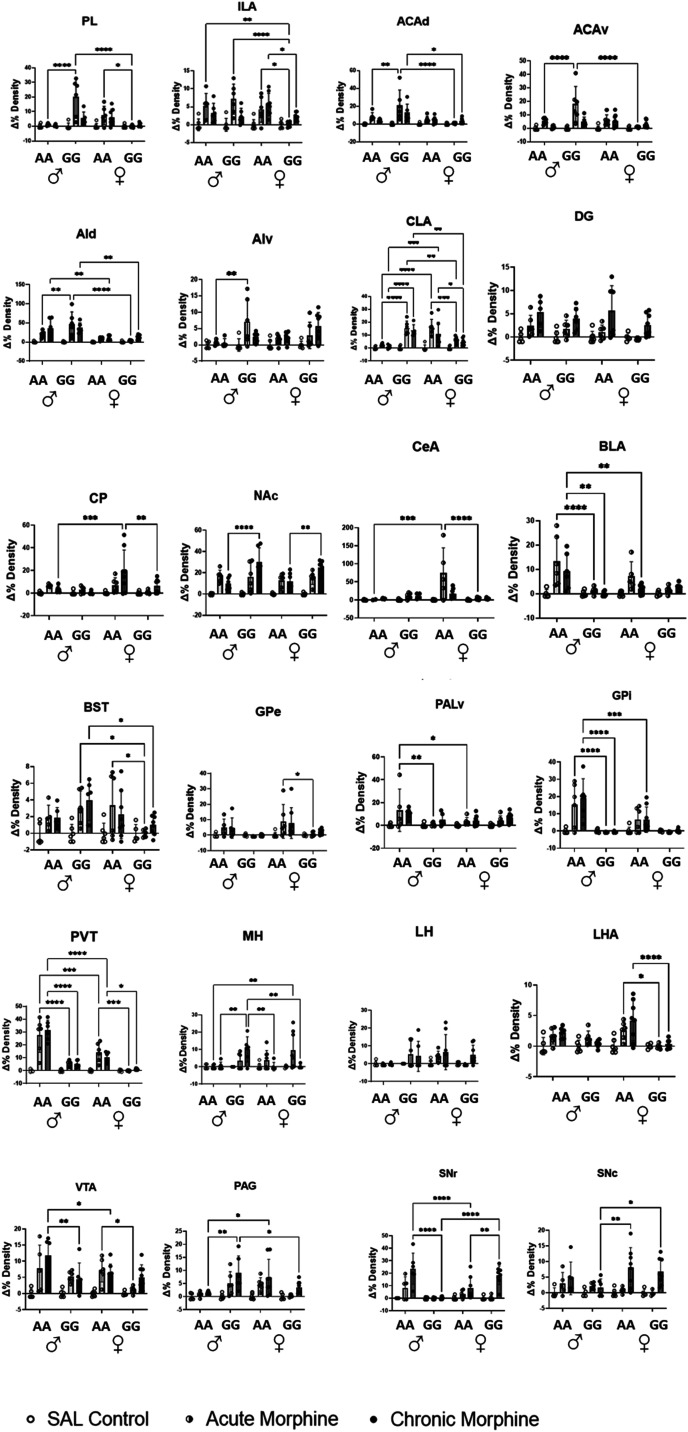
Validation of FOS automated quantification method in randomly selected regions and experimental groups. Each (*X*, *Y*) value in a data point is the detected number of cells (auto, manual) in ROI on a randomly selected slice containing that region. The automatic quantification method effectively captured the relative cell counts compared to manual identification; however, there's interregional wise variance in the overall detection ratio compared to manual (by slope). To avoid this, percentage change (increase over baseline) data was used for interregional comparison instead of raw *c-Fos* density expression (specified in the equation above: detect ratio is different among regions).

Randomly selected regions of FOS expression data from experimental groups were manually quantified with the ImageJ Cell Count Plugin. Pearson correlations were used to assess the degree of similarity between manually quantified and automatically quantified count data for each region (Fig. 2).

### Analysis of FOS expression

Quantified FOS expression data were imported and reformatted through a Python script. FOS-expressing cells per square millimeter were computed and averaged across 2–5 representative sections per brain region. Outliers were identified by the ROUT method with *Q* = 5% and were eliminated. Percent change in FOS expression relative to mean saline FOS expression was calculated for each region. For each ROI, the effects of morphine treatment, genotype, and sex on percent change in FOS expression were analyzed by three-way ANOVA with Tukey's multiple comparison test.

### Analysis of global network properties

Two global graph theory metrics were computed for each FOS correlation network: small-world sigma coefficient and average shortest path length. The small-world coefficient is defined as sigma = *C*/*L*, where *C *= *C*_avg_/*C*_rand_ and *L* = *L*_avg_/*L*_rand_. *C*_avg_ and *L*_avg_ are the average clustering coefficient and average shortest path length of the network, respectively. *C*_rand_ and *L*_rand_ are the average clustering coefficient and average shortest path length of an equivalent random network, respectively. The average shortest path length is defined as the mean number of steps taken along the shortest paths for every possible pair of network nodes. The shortest path between each pair of nodes is that which requires traversing the fewest number of edges. The effects of genotype and treatment on average shortest path lengths were examined by two-way ANOVA with Tukey's multiple comparison test.

### Analysis of local network characteristics

For each network, we computed several region-level graph metrics. To assess region-level contributions to network efficiency, we computed the number of shortest paths that each region intersects. We also computed two centrality metrics: degree centrality and communicability betweenness centrality. Degree centrality represents the proportion of total nodes that are directly connected to each region. Communicability betweenness centrality reflects the total number of weighted walks (not only the shortest paths) connecting every pair of regions in the network (Estrada et al., 2009). For a node u, communicability betweenness centrality is defined as:

$$\mathrm{Betweenness}(u)=\underset{p,q\in \mathrm{Nodes}}{\sum }\frac{\delta (p,q/u)}{\delta (p,q)}(u\neq p,q),\;$$

where δ(p,q/u) is the number of walks intersected by node u and δ(p,q) is the number of walks starting at node p and ending at node q.

### Analysis of anatomically defined meso-structures

To investigate the mesoscale properties of each network, we classified regions according to anatomical groups (meso-structures) defined in the Allen Mouse Brain Atlas: cortex, midbrain, striatum, PAL, and thalamus. We then computed group closeness centrality and group clustering coefficients for each meso-structure. Group closeness centrality is defined as the inverse sum of the minimum weighted distance from regions outside of a group to regions within a group, normalized by the number of regions in the group (Everett and Borgatti, 1999; Zhao et al., 2014) where weights and regions were derived from each FOS correlation network. For a set of nodes V, the group closeness centrality for a group of nodes A is defined as:

$$G(A)=\frac{\left|V-A\right|}{\sum_{v\in V-A}\;\mathrm{dis}t_{A,v}}\setminus \mathrm{dis}t_{A,v}=\mathrm{mi}n_{u\in A}(\mathrm{dis}t_{u,v}),\;$$

A Wilcoxon matched pair signed rank test was used to analyze group closeness centrality with respect to genotype, treatment, and sex difference across the network.

The clustering coefficient represents the proportion of existing connections with neighboring nodes relative to the number of possible connections (Saramäki et al., 2007; Costantini and Perugini, 2014). The clustering coefficient of each node i is defined as:

$$L_{i}=\frac{2*(i\in \Delta )}{\mathrm{degree}(i)(\mathrm{degree}(i)-1)}.$$

The clustering coefficient was computed for each brain region, and group clustering coefficients were then obtained by averaging across the regions within each meso-structure. The effects of genotype, treatment, and sex were assessed using a Friedman test with Dunn's multiple comparison test.

### Network control theory

Within the network control theory framework, neural dynamics are defined by a linear, time-invariant model:

$$\dot{x}(t)=\;Ax(t)+\;B_{k}u_{k}(t),$$

where *x*(*t*) is an *N* × 1 vector representing neural activity (here, percent change in FOS expression, where *N* = 23 brain regions) at a given time point. A is an *N* × *N* adjacency matrix representing the strength of axonal connectivity between each pair of brain regions; here, the mouse structural connectome was derived from open-sourced connectivity data from the Allen Mouse Brain Connectivity Atlas (Oh et al., 2014) with a selective filter including only regions that served as primary injection structures as ROIs. When all brain regions are controlled, B is an *N* × *N* identity matrix with ones along the diagonal and zeros elsewhere. $u_{k}(t)$ is an *N* × 1 vector reflecting the amount of control input into each brain region. By solving for $u_{k}(t)$, we obtain the minimum control energy required for the brain to transition from an acutely exposed to a dependent state.

For each group, the minimum control energy was computed under full control (all brain regions included in the control set) and then recomputed following exclusion of each brain region from the control set. By measuring the increase in minimum control energy induced by removing each node from the control set, we obtained a measure of the influence of each brain region on the total control energy required to transition from an acute to a dependent state.

To perform receptor-weighted control analyses, we extracted μ-, δ-, and κ-opioid receptor gene expression density data from Allen Institute in situ hybridization data (Allen Institute for Brain Science, 2011). Diagonal elements of the control input matrix (B matrix) were weighted to reflect the normalized expression of each of these receptor types. Minimum control energy for the acute to dependent state transition was then recomputed for each receptor distribution, with all regions included in the control set.

## Results

An overview of the experimental design and correlation network analyses is shown in Figure 1*A*.

### Analysis of FOS expression in 24 brain regions revealed heterogeneity between genotype, treatment, and sex

**Figure 3.**
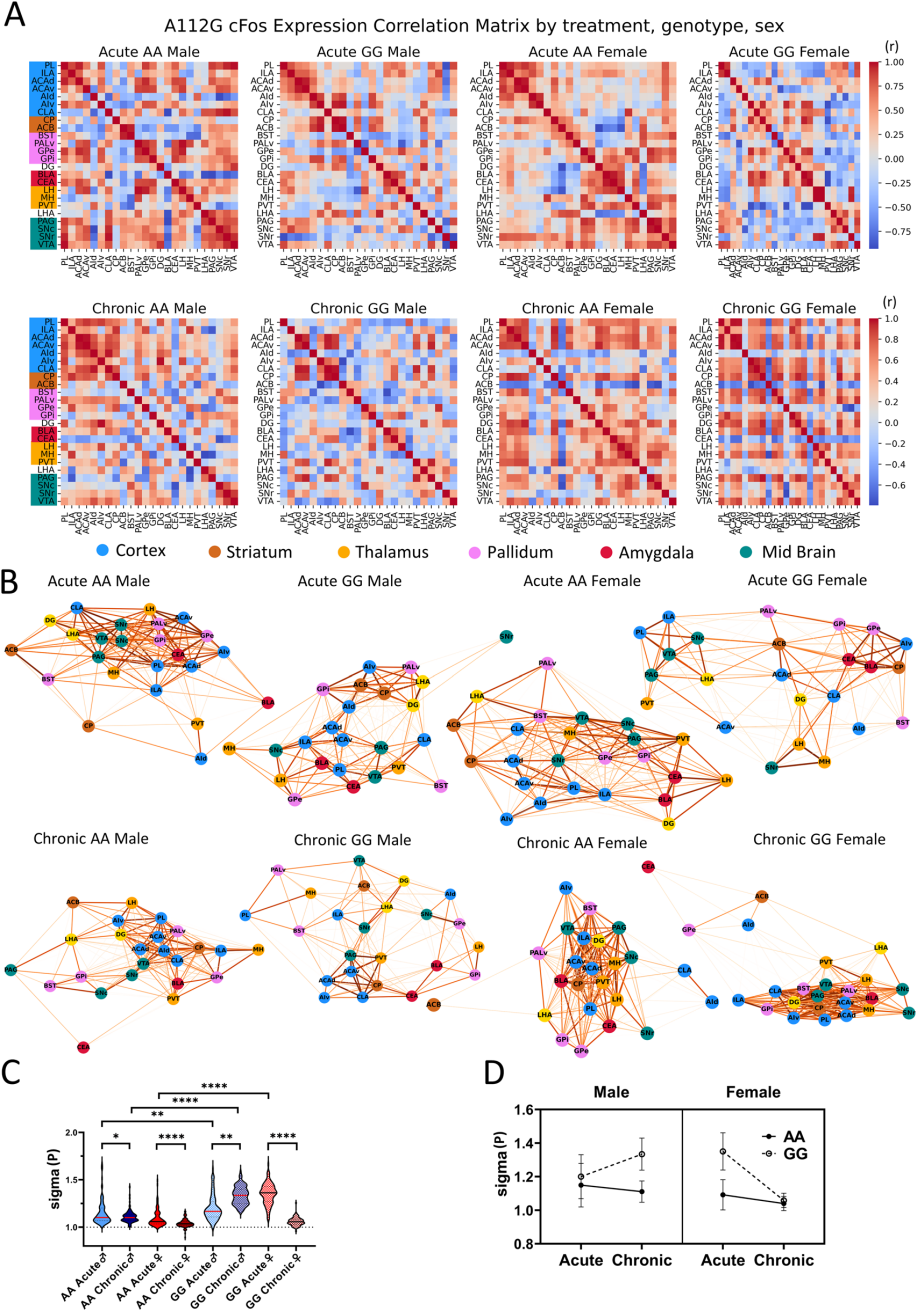
Fos percentage change over baseline of cortical regions in each genotype, treatment, and sex group. PL, prelimbic area; ILA, infralimbic area; ACAd, dorsal anterior cingulate cortex area; ACAv, ventral anterior cingulate cortex area; AId, agranular insular dorsal part; AIv, agranular insular ventral part; CLA, claustrum; CP, caudate putamen; NAc/ACB, nucleus accumbens; CeA, central amygdala; BLA, basolateral amygdala; BST, basal striatal terminalis; GPe, globus pallidus external segment; GPi, globus pallidus internal segment; PALv, ventral pallidum; hippocampus, hypothalamus; PVT, paraventricular nucleus of thalamus; MH, medial habenula; LH, lateral habenula; DG, dentate gyrus; LHA, lateral hypothalamus; VTA, ventral tegmental area; PAG, periaqueductal grey; SNr, substantial nigra reticular part; SNc, substantial nigra compact part. Mean ± SD; Tukey’s post hoc: *p < 0.05, **p < 0.01,***p < 0.001, ****p < 0.0001.

To test the hypothesis that the *Oprm1* SNP alters neuronal activity in response to opioid exposure, we compared FOS expression in AA and GG mice following exposure to saline, acute morphine, or chronic morphine. A validated, automated detection method was used to quantify FOS expression within 24 brain ROIs (Fig. 2). Representative FOS expression is presented in Figure 1*B*. All FOS expression values were normalized to the mean FOS expression of saline-injected mice to compute percent change (Fig. 3). Analysis of percent change in FOS expression by three-way ANOVA revealed significant effects of genotype, treatment, and sex. FOS expression levels differed between AA and GG mice in 12 out of 24 selected brain regions (in cortical, striatal, amygdala, PAL, thalamic, hippocampal, and midbrain regions), while sex differences were observed in 7 out of 24 regions. All 24 regions showed significant treatment effects (Table 1).

**Table 1.** Three-way ANOVA result of 24 regional FOS percentage changes over saline data by genotype, treatment, sex

Structure	Region	Acute versus chronic	Male versus female	AA versus GG
Cortex	ACAd	*F*_(2,65)_ = 15.32****	*F*_(1,65)_ = 16.89***	*F*_(1,65)_ = 3.816
	ACAv	*F*_(2,65)_ = 18.24****	*F*_(1,65)_ = 5.277*	*F*_(1,65)_ = 2.223
	AId	*F*_(2,65)_ = 26.79****	*F*_(1,65)_ = 36.18****	*F*_(1,65)_ = 2.201
	AIv	*F*_(2,64)_ = 8.153***	*F*_(1,64)_ = 0.2413	*F*_(1,64)_ = 10.11**
	CLA	*F*_(2,63)_ = 36.00****	*F*_(1,63)_ = 0.7781	*F*_(1,63)_ = 2.617
	PL	*F*_(2,64)_ = 14.23****	*F*_(1,64)_ = 3.926	*F*_(1,64)_ = 3.096
	ILA	*F*_(2,64)_ = 19.52****	*F*_(1,64)_ = 2.656	*F*_(1,64)_ = 3.649
Striatum	CP	*F*_(2,65)_ = 7.224**	*F*_(1,65)_ = 4.741*	*F*_(1,65)_ = 7.760**
	NAc	*F*_(2,65)_ = 55.27****	*F*_(1,65)_ = 0.8211	*F*_(1,65)_ = 13.59***
Pallidum	BST	*F*_(2,61)_ = 9.292***	*F*_(1,61)_ = 2.236	*F*_(1,61)_ = 0.2149
	PALv	*F*_(2,63)_ = 9.154***	*F*_(1,63)_ = 1.922	*F*_(1,63)_ = 3.252
	GPe	*F*_(2,63)_ = 3.657*	*F*_(1,63)_ = 2.433	*F*_(1,63)_ = 11.10**
	GPi	*F*_(2,63)_ = 7.994***	*F*_(1,63)_ = 6.450*	*F*_(1,63)_ = 37.74****
Amygdala	CeA	*F*_(2,56)_ = 6.016**	*F*_(1,56)_ = 5.838*	*F*_(1,56)_ = 4.716*
	BLA	*F*_(2,62)_ = 11.48****	*F*_(1,62)_ = 3.492	*F*_(1,62)_ = 20.92****
Thalamus	PVT	*F*_(2,56)_ = 33.20****	*F*_(1,56)_ = 30.49****	*F*_(1,56)_ = 82.43****
	LH	*F*_(2,64)_ = 3.833*	*F*_(1,64)_ = 0.8397	*F*_(1,64)_ = 0.3215
	MH	*F*_(2,63)_ = 4.635*	*F*_(1,63)_ = 1.510 × 10^−5^	*F*_(1,63)_ = 7.935**
Midbrain	VTA	*F*_(2,62)_ = 25.41****	*F*_(1,62)_ = 3.947	*F*_(1,62)_ = 12.28***
	PAG	*F*_(2,62)_ = 14.41****	*F*_(1,62)_ = 0.01579	*F*_(1,62)_ = 0.4960
	SNr	*F*_(2,62)_ = 32.53****	*F*_(1,62)_ = 0.05843	*F*_(1,62)_ = 8.911**
	SNc	*F*_(2,60)_ = 16.58****	*F*_(1,60)_ = 0.8094	*F*_(1,60)_ = 1.645
Hippocampus	DG	*F*_(2,62)_ = 19.54****	*F*_(1,62)_ = 1.591	*F*_(1,62)_ = 3.357
Hypothalamus	LHA	*F*_(2,63)_ = 9.744***	*F*_(1,63)_ = 0.4553	*F*_(1,63)_ = 18.15****

* *p* < 0.05.

** *p* < 0.01.

*** *p* < 0.001.

**** *p* < 0.0001.

**Software 1.** All code is available on GitHub: git@github.com/GoogleXie/Oprm1-cFos-Connectivity.git.

### FOS correlation networks are small-world networks

**Figure 4.**
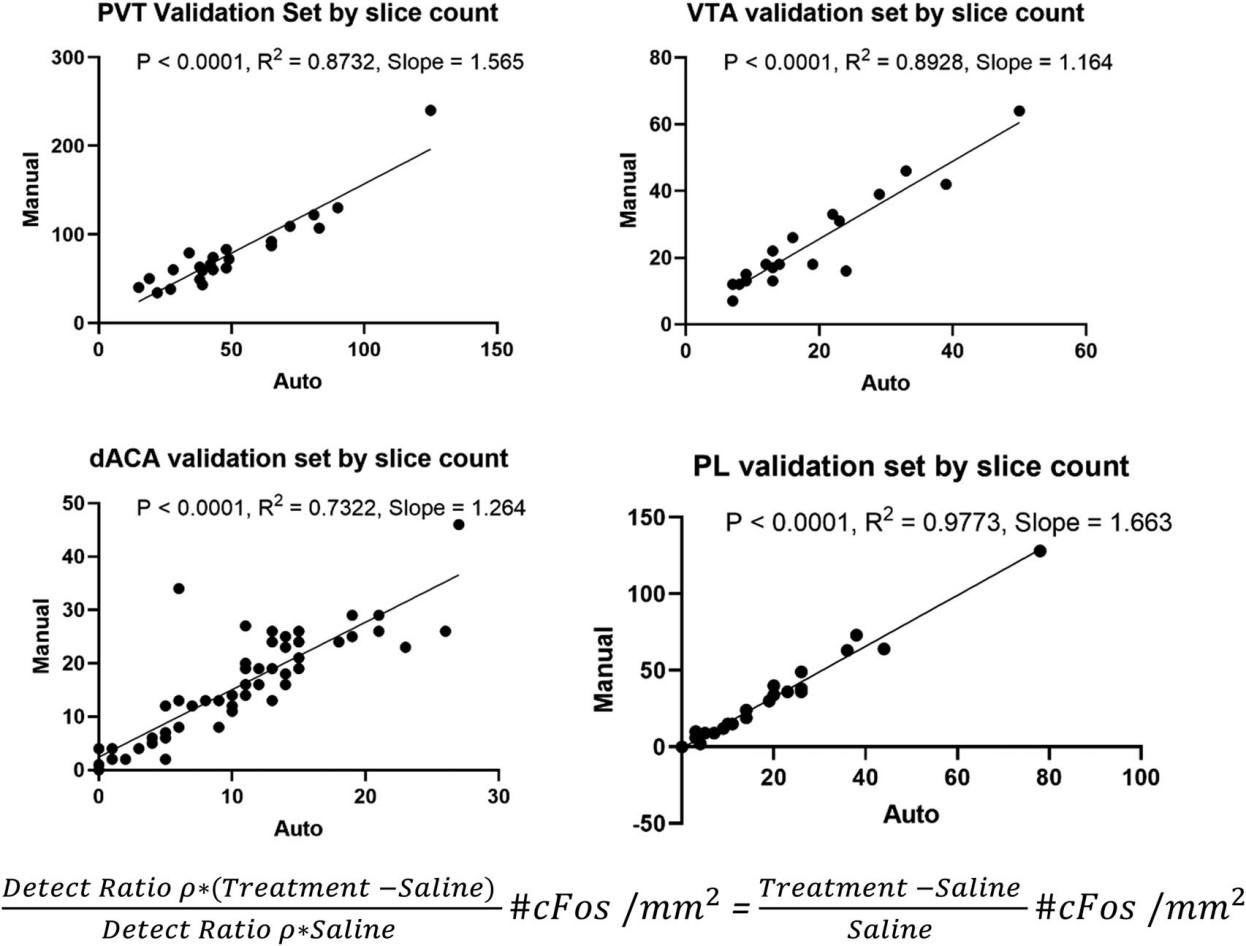
Interregional characteristics of A112G FOS percentage change. ***A***, Correlation matrix based on FOS percentage change of expression over baseline and grouped by macro-anatomical regions in acute morphine exposure (top row) and chronic morphine exposure (bottom row). Each grid in the matrix represents the Pearson correlation coefficient between two regions. ***B***, Correlation network (2D, force-directed) depicts positive pairwise correlations where correlation strength decides the dragging force between two regions. ROIs are colored according to their mesoscale structure (blue = cortex, brown = striatum, gold = thalamus, red = amygdala, violet = pallidum, green = midbrain, yellow = hippocampus and hypothalamus). ***C***, Three-way ANOVA on the small-world coefficient of bootstrapped correlation network showed significant treatment, sex, and genotype effects. Tukey’s post hoc multiple comparison test showed significant between-group difference. Small-world coefficient sigma, calculated by the avg clustering coefficient and avg shortest path length of the network with respect to random graphs (*N* = degree of correlation network). ***D***, All correlation networks show sigma > 1: small-world property is maintained (mean ± SD; **p < 0.01, ****p < 0.0001).

After quantifying the region-specific effects of the *Oprm1* genotype on FOS expression, we generated FOS correlation networks for each experimental group. We first generated a correlation matrix representing pairwise Pearson correlation coefficients for each genotype, treatment, and sex condition (Fig. 4*A*). Correlation network graphs for each experimental group were generated with nodes representing brain regions and edges corresponding to pairwise positive correlations in FOS expression between regions. FOS correlation networks were plotted such that the edge lengths equal the log inverse of the correlation strength to map strong positively correlated regions (Pearson *R* ∼1) into close neighborhoods (distance ∼0) and weakly correlated regions (Pearson *R* ∼0) further apart (distance ∼∞) (Fig. 4*B*).

We used graph theory metrics to characterize the topological properties of FOS correlation networks following acute and chronic opioid exposure in AA and GG mice (Simpson et al., 2021). We first explored whether opioid dependence affects the small-world properties of our FOS correlation networks. Small-world network characteristics are evident if most of the brain regions (nodes) are not directly connected to each other, but rather are connected through only a few or “small” number of indirect connections. Significant changes in small-world properties have been observed in rs-fMRI connectivity networks in individuals with OUD (Yuan et al., 2010; Jiang et al., 2013; Wang et al., 2015). Specifically, a lower small-world scale (i.e., sigma coefficient) was observed in individuals with heroin dependence (Jiang et al., 2013) or following abstinence from heroin use (Yuan et al., 2010).

The FOS correlation networks for all experimental groups showed a sigma coefficient significantly above 1 after opioid exposure (ANOVA: *p* < 0.0001, DF = 99) and were thus classified as small-world networks. Significant effects of genotype (*F*_(1,792)_ = 427.1, *p* < 0.001), sex (*F*_(1,792)_ = 90.44, *p* < 0.001), and treatment (*F*_(1,792)_ = 86.87, *p* < 0.001) on small-world sigma were observed. Both male and female AA mice showed decreases in small-world sigma following chronic opioid exposure [Šídák's post hoc, AA male, *p* = 0.0452%, 95% CI = (0.0004341 to 0.07681); AA female, *p* = 0.0013%, 95% CI = (0.01358 to 0.08996)]. Sigma coefficients were higher among GG mice relative to AA mice across most conditions and were markedly higher in the chronic state than the acute state in GG males. Interestingly, the opposite pattern was observed in GG females: the sigma coefficient was significantly lower in the chronic relative to the acute state [GG male, *p* < 0.0001%, 95% CI = (−0.1723 to −0.09593); GG female, *p* < 0.0001%, 95% CI = (0.2539 to 0.3303); Fig. 4*C*,*D*].

### Local network efficiency is elevated in GG mice during morphine administration

**Figure 5.**
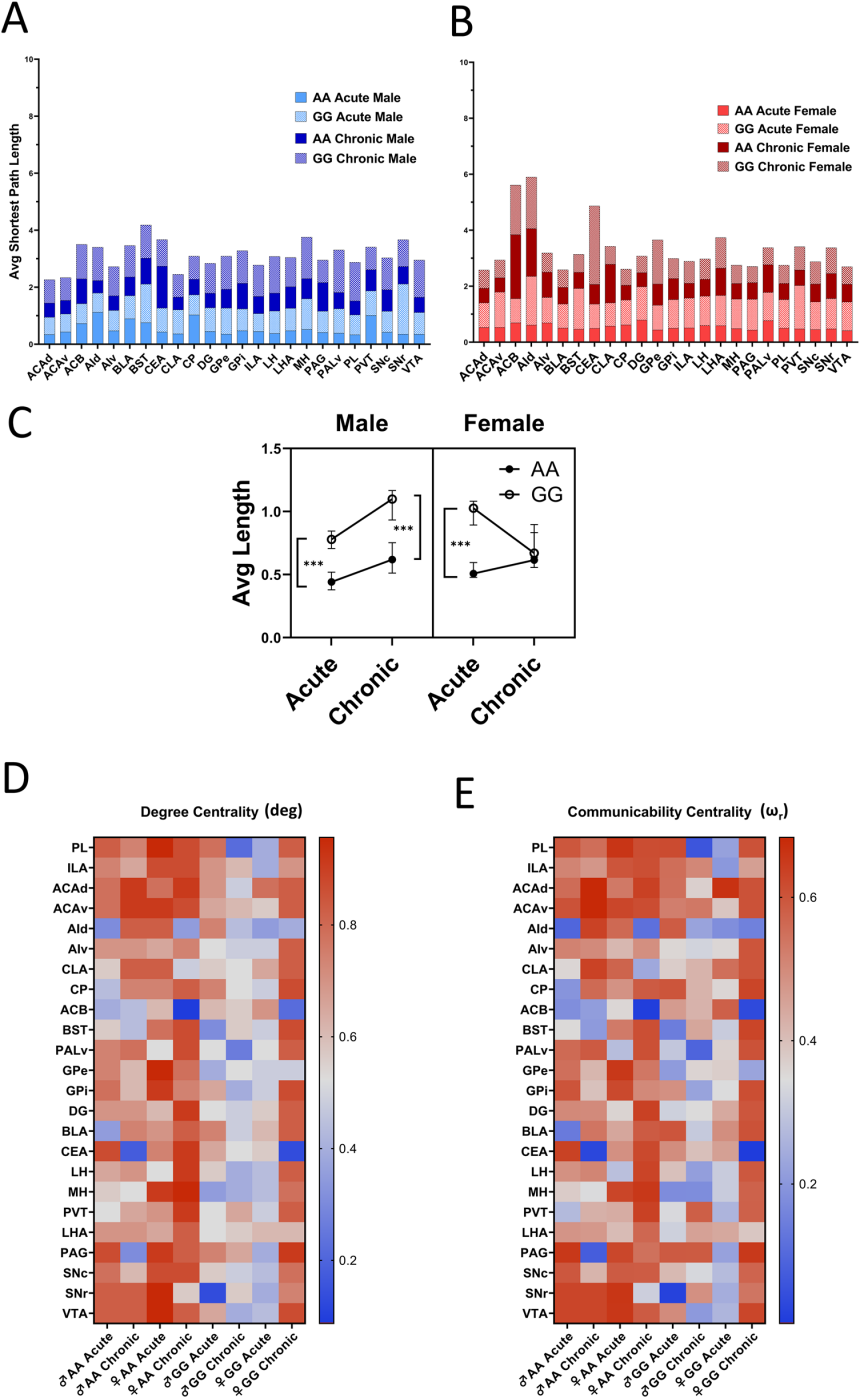
Regional analysis of FOS network. ***A,B***, Shortest path length plotted for each region by genotype and treatment in male and female. Average shortest path length pairwise comparison between each experimental condition by nonparametric Friedman test with Dunn's multiple comparison. ***C***, Average shortest path length comparison between each experimental condition by nonparametric Friedman test with Dunn's multiple comparison (median ± 95% CI, *n* = 24; ***p < 0.001). ***D***, Degree centrality calculating the fraction of the total possible connection for each region in the correlation network. Friedmann test with Dunn's multiple comparison by genotype, treatment, and sex. ***E***, Communicability betweenness centrality calculated by all weighted paths connecting pairs of regions, summarized by regions in correlation network. Friedmann and Dunn's multiple comparison test by genotype, treatment, and sex (median ± 95% CI; *p < 0.05, **p < 0.01, ***p < 0.001).

The significant difference in the Sigma coefficient of GG mice compared to AA mice motivated us to further interrogate genotype effects on network connectivity. Small-world networks typically have relatively high clustering (densely connected networks) and relatively low average shortest path lengths; each of these properties serve to reduce wiring energy cost (Latora and Marchiori, 2001; Bassett and Bullmore, 2017). Therefore, we wanted to examine these two properties separately. The shortest path length represents the average number of edges that must be traversed in order to move between every possible pair of nodes in a network and is an indicator of local network efficiency (Latora and Marchiori, 2001). We found that the average shortest path length varies across regions, genotype, and sex (Fig. 5*A,B*). The average shortest path length was significantly higher in GG mice compared to AA mice except in the female chronic exposure group (Fig. 5*C*). As the shortest path length measures the efficiency of information transport in a network, the reduction in the shortest path length in GG mice suggested a stronger local network efficiency in the FOS correlation networks of GG mice following either acute or chronic morphine administration.

### Genotype- and sex-specific effects of morphine on degree centrality

To further investigate the impact of genotype and treatment on network connectivity, we measured degree centrality (unweighted; where matrix elements are either 0, connection does not exist, or 1, connection exists; Latora and Marchiori, 2001). Unweighted degree centrality is a measure of the number of the number of connections each node makes to other nodes. In contrast to average path length, which reflects network efficiency, degree centrality reflects the relative importance of a node in fostering communication; those with higher degree centrality are more strongly connected to the rest of the network. We found that GG males showed significantly lower degree centrality in the morphine-dependent state relative to the acute state, and lower degree centrality overall compared to AA males, suggesting lower interregional connectivity (Friedman: *p* < 0.001, *Q* = 68.87; Dunn's post hoc: *p* = 0.007, *Z* = 3.447). Degree centrality is also lower in GG females relative to AA females in the acute opioid-naive state (*p* < 0.001, *Z* = 4.95) but not in the chronic dependent state (Fig. 5*D*).

We also examined communicability betweenness centrality. Communicability betweenness centrality reflects the number of weighted paths from one region that are connected to all other regions in the network and thus serves as a measure of the extent to which one brain region acts as an intermediary between other brain regions. We calculated the number of direct connections (weighted by correlation strength) between every pair of regions and observed a similar pattern compared to degree centrality (*p* < 0.001, *Q* = 32.42). GG males showed a nonsignificant trend toward lower communicability betweenness centrality during chronic exposure to morphine compared to AA males (*p* = 0.07, *Z* = 2.77). Following acute morphine administration, communicability betweenness centrality was lower in GG females compared to AA females (*p* = 0.01, *Z* = 3.241). However, there was no effect of genotype on communicability betweenness centrality in opioid-dependent females (*p* > 0.99, *Z* = 0.4714) (Fig. 5*E*). Overall, we observe a similar pattern between weighted and unweighted centrality measures, which suggests that the major impact of the *Oprm1* SNP is on the number of connections rather than the strength of connections within the respective networks.

### Meso-structure segregation differs between sex and genotype

**Figure 6.**
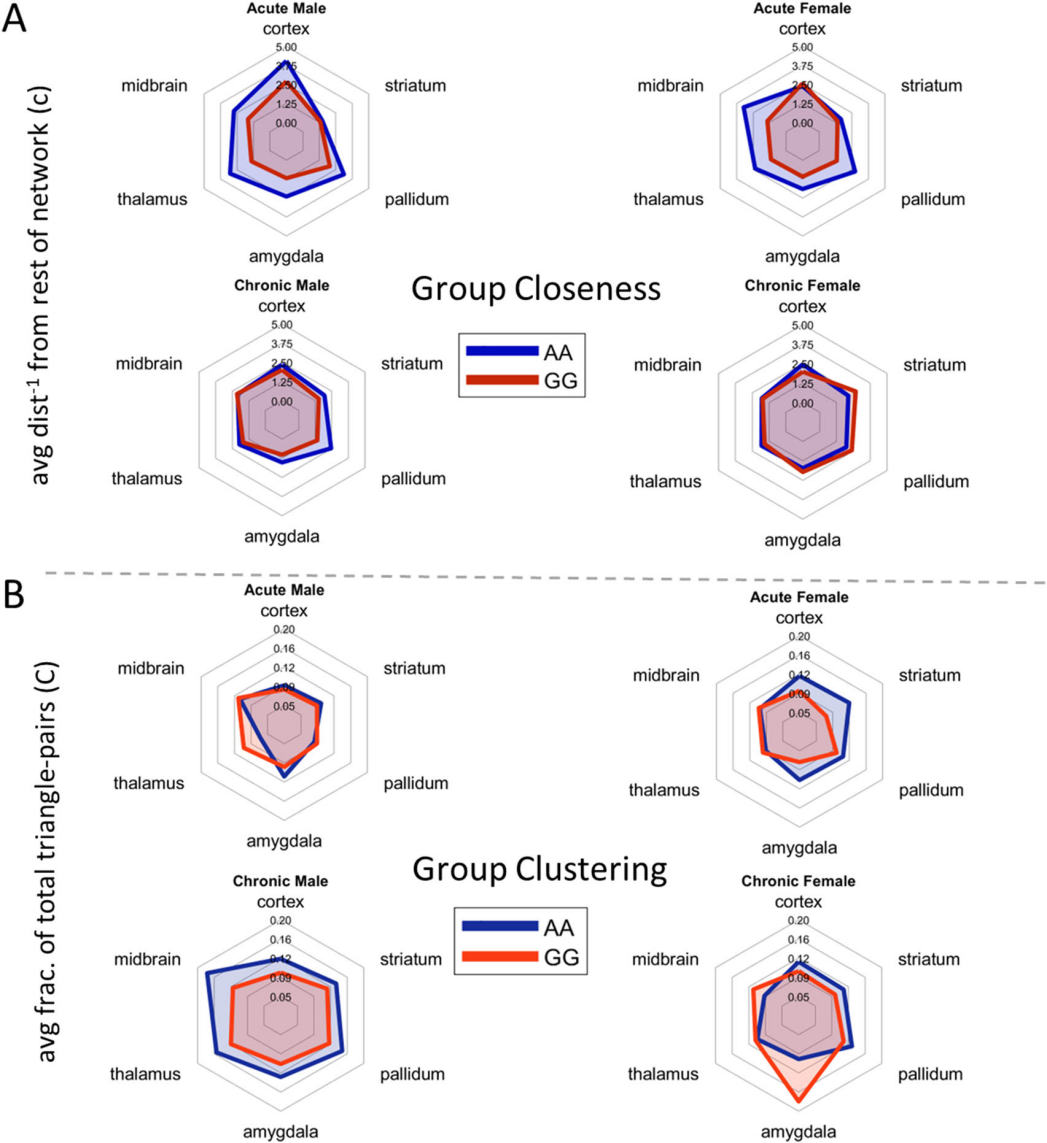
Analysis of correlation network meso-structures. ***A***, Group closeness centrality reflects the average distance (inversed) of a meso-structure (e.g., cortex) from the rest of the network. High group closeness centrality reflects strong connectivity with the network. ***B***, Group clustering coefficient reflects the average fraction of total possible triangle-pairs of regions that a structure intersects (unweighted). High clustering coefficients indicate strong within-group connectivity.

Alterations in clustering between and within mesoscale structures of regions could underlie differences in small-world network properties. We analyzed the connectivity of six anatomically defined mesoscale structures (cortical, striatal, amygdala, PAL, thalamic, and midbrain regions) with the rest of the network. Group closeness centrality was 1.5- to twofold higher in AA males compared to GG males in all regions except the striatum and two- to threefold higher in AA females compared to GG females in all regions except the striatal and cortical regions. Higher group closeness centrality in these brain structures suggests stronger connectivity with the remainder of the brain in AA mice compared to GG mice. Meanwhile, lower group closeness centrality observed in GG relative to AA mice after acute exposure was not evident after chronic exposure to opioids (Fig. 6*A*). Overall, group closeness centrality declined in AA mice, but not in GG mice, suggesting that these brain structures maintain connectivity strength in GG mice during the transition from opioid-naive to an opioid-dependent state.

Next, we analyzed the group clustering coefficient in the same six mesoscale structures. We observed an increase in clustering coefficient within meso-structures only in AA males during the transition from the opioid-naive to the opioid-dependent state, leading to a 1.2- to 1.4-fold higher group clustering coefficient compared to GG males in the chronic state. In the acute state, we observed 1.3- to 1.7-fold higher clustering in the cortex, striatum, and amygdala of female AA mice compared to female GG mice. This difference diminished after chronic exposure. In contrast, GG females showed a 1.8 and 1.2-fold higher clustering coefficient in amygdala and midbrain, respectively, following opioid exposure (Fig. 6*B*). Together, these data indicate that genotype influences segregation between meso-structures in the opioid-naive state but influences clustering within meso-structures in the opioid-dependent state.

### GG females require an outsized amount of energy input to transition to a morphine-dependent brain state

**Figure 7.**
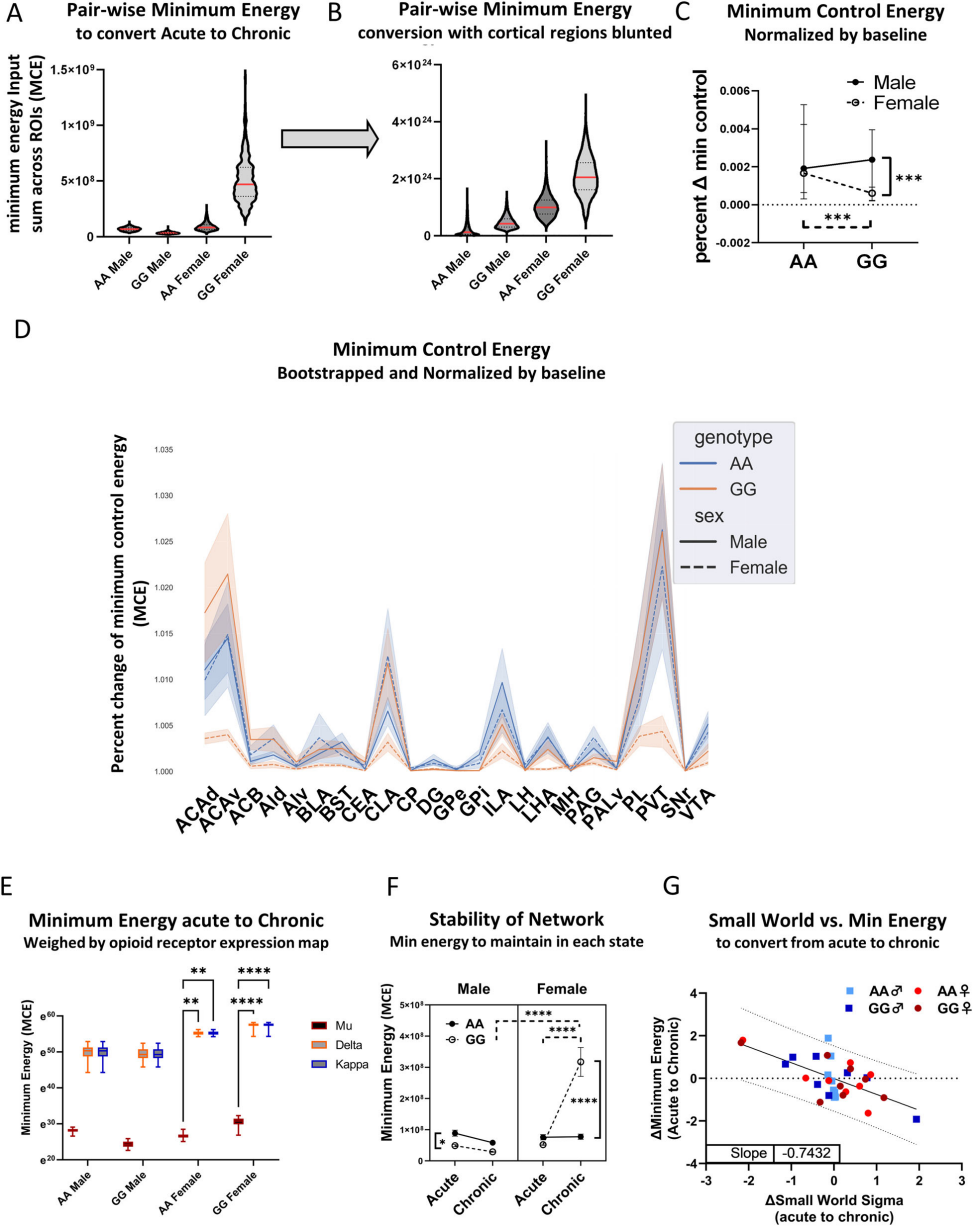
Brain state transition analysis. ***A***, Minimum control energy required to transition from an acute morphine-exposed state to a chronic dependence state when all regions are controlled for each genotype and sex after bootstrapping (red line = median). GG females showed significantly higher minimum energy requirements to transition to an opioid-dependent state compared to AA females (ANOVA, *R*^2^ = 0.7753, *F*_(3,159996)_ = 184016, *p* < 0.0001; Tukey, *p* < 0.0001, *Q* = 803.2). GG males showed lower minimum energy requirements compared to AA males (Tukey, *p* < 0.0001, *Q* = 64.57) ***B***, Minimum control energy required to transition from acute morphine to morphine-dependent brain state after suppressing input to cortical regions (dACA, vACA, AId, AIv, CLA, PL, ILA) for each genotype and sex. ***C***, Percent increase in minimum control energy following suppression of control input to each region. ***D***, Minimum control energy for state transition after weighting control inputs by expression density map of μ-, δ-, and κ-receptors. ***E***, Minimum control energy required to maintain each brain state (median ± 95% CI; *p < 0.05, ***p < 0.001, ****p < 0.0001). ***F***, Pearson correlation of minimum control energy required to transition from an acute to a chronic state with small-world coefficient sigma on bootstrapped (*N* = 8) and *z*-transformed data. A significant negative correlation was identified (mean ± 95% prediction bands).

Although graph theory analyses provide important insight into the impact of morphine exposure on functional interactions between brain regions, in the interest of better understanding how the *Oprm1* A118G SNP influences addiction liability, it will be important to gain a better understanding of the modulatory influence of this SNP on brain network dynamics during the *transition* from initial exposure to dependence. To examine this question, we simulated network dynamics using a network control theory approach. Network control theory stipulates that the brain's structural connectome constrains its repertoire of neural activity states (Gu et al., 2015). In our model, FOS expression levels across brain regions at each time point represented brain states, and the structural connectome was constructed using open-resource data from the Allen Institute (Allen Institute for Brain Science, 2011). When control input is provided to each of our 23 ROIs (1 region was eliminated because it was not a primary injection site), we found that an outsized minimum control energy is needed to transition from the acute morphine-exposed state to the chronic morphine-dependent state in GG females (AA male = 6.01 × 10^7^, GG male = 2.59 × 10^7^, AA female = 8.92 × 10^7^, GG female = 5.54 × 10^8^). We then used bootstrapping (*n* = 200) to generate a distribution of FOS expression vectors and calculated the minimum control energy required to transition between pairs of resampled neural activity states. Each resample was treated as a neural activity state; thus, 200 acute brain states and 200 chronic brain states were simulated (resulting in 200^2^ possible state transitions for each genotype and sex). GG females showed significantly higher minimum control energy relative to other groups (mean, AA male = 6.9 × 10^7^, GG male = 3.5 × 10^7^, AA female = 9.1 × 10^7^, GG female = 5.1 × 10^8^) (Fig. 7*A*).

### Suppression of control input to cortical regions minimizes genotypic differences in the minimum control energy required to transition to the dependent state

To identify brain regions that drive the transition to a dependent brain state, we computed the relative influence of each region on the minimum control energy required for this transition (represented by the increase in minimum control energy input following the removal of each region from the control set). By this analysis, we determined that the dACA, vACA, CLA, ILA, PL, and PVT (hereafter referred to as “target regions”) strongly influence minimum control energy in males of both genotypes and in AA females. In GG females, we observed less variation in the relative influence of each region on minimum control energy (Fig. 7*C*).

Suppressing the control input to all target regions reduced the difference in minimum control energy requirement between GG and AA females from 6.3-fold (5.1 post-bootstrap and pairwise comparison) to 1.9-fold. This reduction is significantly greater than that induced by randomly suppressing control input to other equally sized subsets of regions (*M* = 6.428, SD = 8.932, *t*_(99)_ = 4.846, *p* < 0.0001). In other words, control input to cortical regions is the main factor contributing to the genotypic difference in minimum energy required to transition to an opioid-dependent state (Fig. 7*B*).

Next, we examined the influence of receptor distribution on the minimum control energy required to transition to the chronic state in our opioid dependence model. Opioid receptor (mu, delta, kappa) expression density maps of 24 ROIs were computed based on in situ hybridization imaging data from the Allen Institute (Allen Institute for Brain Science, 2011). We recalculated the minimum control energy requirement for the transition between naive and dependent brain states with control inputs weighted according to the distribution of opioid receptor expression in each region. We found that weighting control inputs according to μ-opioid receptor expression levels enabled transitioning to the dependent state with much lower minimum control energy than when the inputs were weighted by the transcript levels of the other two opioid receptors (ANOVA, *p* < 0.0001; Tukey’s post hoc, *p* < 0.0001; mean diff mu vs delta, mu vs kappa, −2.884^24^). Differences in genotype and sex were maintained (ANOVA, *p* < 0.0001; *F* = 409.6), as would be expected given that the same input for opioid receptor expression was used for each genotype and sex—a notable caveat. Of interest, there is no correlation between FOS activation with μ-expression density (data not shown). Meanwhile, no significant difference was observed when using δ- and κ-weighted minimum energy input (Tukey’s post hoc, *p* > 0.99; mean diff delta vs kappa, 3.22^9^; Fig. 7*D*).

To assess the stability of each brain state, we computed the minimum control energy required to maintain each state (i.e., initial and final states are represented by the same neural activity vectors). We found that GG females required significantly higher minimum control energy to maintain their dependent state compared to their acute state (ANOVA and Šídák's post hoc, *p* < 0.0001) and compared to the energy required by AA females in the chronic state (Šídák's post hoc, *p* < 0.0001). Meanwhile, GG males required lower minimum energy to maintain the acute state compared to AA males (Šídák's post hoc, *p* = 0.0139). The finding that GG females require higher minimum energy both to reach and maintain an opioid-dependent brain state suggests that this state is relatively unstable (Fig. 7*E*).

### The minimum control energy required to transition to a dependent state is correlated with the change in small-world properties

Since we observed differences in both functional network properties and minimum energy requirements for the transition to a dependent brain state in GG mice, we wanted to explore the potential link between them. We observed that GG females showed a significant decrease in small-world sigma in the dependent state and also required the most control energy input for the transition from an acute to a dependent state. In contrast, GG males showed increases in small-world properties and had the lowest minimum control energy requirement. We hypothesized that changes in the small-world sigma of the opioid-induced FOS correlation network are related to the minimum control energy required to transition from the acute to the dependent state. We transformed bootstrapped (*N* = 8) small-world scores (chronic—acute) and minimum energy requirements of each group to a *Z*-score. As predicted, we found a significant negative correlation between the change in small-world sigma and minimum energy requirements to transition from the acute to the dependent state across experimental groups [*R*^2^ = 0.4151, *F*_(1,30)_ = 21.29, *p* < 0.0001, Slope 95% CI = (−1.072 to −0.4142); Fig. 7*F*].

## Discussion

OUD is associated with enduring changes in brain plasticity. This plasticity can occur at the level of individual neurons which can alter communication between neurons and ultimately impact whole brain connectivity. In the past few years, there has been growing interest in network neuroscience and connectivity-based approaches to understanding OUD. Decreased connectivity between regions of the anterior default mode network (DMN) has been identified in individuals with SUD compared to controls (Ma et al., 2015; Tang et al., 2016), and an inverse correlation between DMN connectivity and heroin cravings has been shown, such that lower connectivity is associated with stronger cravings (Li et al., 2016). A limitation inherent to these and many clinical studies is the inability to distinguish state from trait markers of OUD; in other words, differences that result from chronic opioid exposure versus those that may predispose an individual, under certain circumstances, to developing OUD. One well-established trait marker for OUD is the *OPRM1* A118G SNP. While genetic factors account for up to 80% of disease risk for opioid dependence (Goldman et al., 2005; Agrawal and Lynskey, 2008), how specific genetic variants modulate the brain to cause behavioral changes is unknown.

The *Oprm1* A112G mouse model possesses loss-of-function phenotypes analogous to those reported in human studies, such as decreased expression, reduced morphine-mediated antinociception, and decreased hedonic reward (Mague et al., 2009; Zhang et al., 2015, 2020). However, decreases are not present in all morphine-mediated behaviors, suggesting that the alterations in response to opioids can be dependent on other factors, such as altered neural networks. Regional and network-level changes in *c-Fos* gene expression and protein levels have been identified in a wide range of studies in response to opioids, cocaine, amphetamine, ethanol, and nicotine (Piechota et al., 2010; Yu and Sharp, 2012; Charbogne et al., 2017; Kimbrough et al., 2020; Nawarawong and Olsen, 2020; Carrette et al., 2023; Roland et al., 2023; Stefaniuk et al., 2023). Specifically, *c-Fos* expression is increased in caudoputamen (CP), nucleus ACB, and the VTA following acute morphine as well as heroin self-administration (Piechota et al., 2010; Charbogne et al., 2017). Forebrain-specific deletion of µ-opioid receptors led to reduced or blunted induction of c-Fos in CP and ACB (Charbogne et al., 2017). Thus, µ-opioid receptors influence neural network activities across multiple brain circuits. While many studies investigating substance use and abuse focus on subcortical regions, brain-wide c-fos network analysis has illuminated relationships between network-level changes with relapse risk (Kimbrough et al., 2021).

In the present study, we used graph theory metrics to assess the impact of the *Oprm1* A118G SNP on neural connectivity at a brain-wide scale. Across all conditions, FOS correlation networks displayed small-world network properties, which are typical of most mammalian brain networks (Latora and Marchiori, 2001; Srinivas et al., 2007; Stam et al., 2007; Yuan et al., 2010; Wang et al., 2015; Bassett and Bullmore, 2017; Liao et al., 2017). Neural networks are mainly built upon two principles: enhancing interregional communication efficiency (clustering) and reducing the cost of forming interregional connections (path length) (Bassett and Bullmore, 2017). Small-world networks are known for their high level of clustering and having very short path lengths between the nodes of the network. While all mice showed properties associated with small-world networks, all GG animals showed greater efficiency with the exception of the GG females in the dependent state, which more closely resembled the AA mice of both sexes.

Taken together, we found that even though GG females have less communication efficiency compared to AA in the opioid-naive state, similar patterns of clustering exist both between and within meso-structures in the opioid-naive and opioid-dependent state. Overall, a consistent pattern emerges in GG females across regional and meso-structure analyses pointing to a better balance between communication efficiency and cost of connectivity which is dysregulated following chronic morphine exposure.

To identify brain regions that drive network-level changes in the transition from opioid-naive to dependent state, we employed a network control theory approach (Gu et al., 2015; Karrer et al., 2020), which has previously been used to identify regions that drive opioid-mediated brain state transitions (Brynildsen et al., 2020). By representing the brain's structural connectome with data from the Allen Mouse Brain Connectivity Atlas (Oh et al., 2014), we computed the minimal input energy required to transition from an opioid-naive to an opioid-dependent brain state when control input is given to all regions in the network. Our results indicated a more than sixfold increase in average minimum control energy (5.2 after pairwise computation of bootstrapped samples) to convert to the dependent state compared to all other experimental groups. Taken together with our prior findings, the significantly higher-energy requirement for the transition from a naive to a dependent state in GG females indicates that GG females experience a more drastic change in both functional network features and neural dynamics after chronic morphine administration.

We also quantified the relative influence of each region on the energetic cost of transitioning to an opioid-dependent brain state. For each state transition, the minimum control energy was first calculated under full control (meaning control input is delivered to all regions) and then recalculated following suppression of each region (control input is delivered to all regions except the suppressed region). The minimum control energy computed following the suppression of input to each region was normalized to minimum control energy in the full control condition to compute percent change. Across all experimental groups, the increase in minimum control energy was highest following the suppression of input to target regions (the dACA, vACA, CLA, ILA, PL, and PVT). All except one of these target regions are cortical regions and are considered to have a fundamental role in behaviors associated with “anticipation/preoccupation” according to the brain disease model of addiction (U. S. Department Human Services, Office of the Surgeon General, 2016).

Suppressing input to target regions in GG females minimally impacted the control energy required to transition to an opioid-dependent state relative to other groups. To test the hypothesis that differences in energy cost in GG females relative to AA females may be accounted for by input to target regions, we recalculated the minimum control energy following suppression of control input to all six of these regions. Suppressing input to the six target regions significantly reduced the difference in energy cost between GG females and AA females (sixfold to 1.9-fold). We verified the regional specificity of this effect by comparing it with the difference in energy cost induced by suppressing control input to randomly selected, equally sized groups of regions and found that the impact of these target regions was significantly greater. These data suggest that relatively lower engagement of cortical regions may counteract the formation of the dependent state in the presence of the G allele.

To incorporate the pharmacological site of action of morphine in our network control theory analysis, we extracted opioid receptor expression density maps from Allen Institute ISH data (Allen Institute for Brain Science, 2011) and weighted the control inputs such that they reflect receptor expression levels in each region. We demonstrate that weighting control inputs according to the distribution of µ-opioid receptors enables a lower-cost transition to an opioid-dependent state. The relatively low-energy state transition induced by the distribution of µ-opioid receptors relative to kappa or delta is consistent with the known role of µ-opioid receptors in mediating morphine responses (Matthes et al., 1996; Jordan et al., 2000). Specifically, previous findings indicate that µ-opioid receptors, but not δ-opioid receptors, mediate morphine reward on a network level (David et al., 2007). On the other hand, the relatively high minimum energy requirements suggest that the dependent state may be more difficult to reach in GG females. Consistent with this finding, we also determined that the opioid-dependent state in GG females was relatively unstable; in other words, significantly more control energy is required to maintain the opioid-dependent brain state.

One of the major findings from this work is that GG females have a relatively efficient network behavior in the opioid-naive state that is disrupted in the dependent state. The lack of a stable network during dependence could be protective. However, a major caveat of these studies is the mode of opioid exposure, which was investigator-administered rather than self-administered. Self-administration better reflects human drug-seeking behavior (U. S. Department Human Services, Office of the Surgeon General, 2016). Importantly, our escalating chronic morphine exposure paradigm in the mouse model does effectively capture the physiological dependence associated with OUD.

While most existing opioid treatments are focused on receptor targets, our identification of key cortical regions associated with drug dependence suggests that novel noninvasive approaches could be used for therapeutic intervention. Repetitive transcranial magnetic stimulation (r-TMS) or transcranial alternating current stimulation (TACS) have been used as effective modulators of brain connectivity (Clancy et al., 2018; Beynel et al., 2020; Kar et al., 2020). In this study, we identified neural substrates that could be targeted to alter connectivity and prevent the establishment of the dependent brain state (Xiu et al., 2014). To date, this is the first analysis of network alterations due to the *Oprm1* A118G SNP. These data establish the groundwork for future studies using novel techniques such as chemogenetic and optogenetic approaches to identify causal mechanisms underlying neural connectivity in the whole brain.
